# Ultrasonographic changes following pyeloplasty in children with ureteropelvic junction obstruction: a prospective observational before-after cohort study

**DOI:** 10.1097/MS9.0000000000005679

**Published:** 2026-09-16

**Authors:** Farnoush Izadfar, Reza Abbasioun, Ahmad Mohammadipour, Sara Saadat, Mostafa Abrishami, Sayed Morteza Fayez, Mohammad Mahdi Alvandi Fard, Khashayar Atqiaee

**Affiliations:** a Department of Pediatric Surgery, Akbar Children’s Hospital, Faculty of Medicine, Mashhad University of Medical Sciences, Mashhad, Iran; b Department of Pediatric Urology, Akbar Children’s Hospital, Faculty of Medicine, Mashhad University of Medical Sciences, Mashhad, Iran; c Department of Pediatric Nephrology, Mashhad University of Medical Sciences, Mashhad, Iran; d Student Research Committee, Faculty of Medicine, Mashhad University of Medical Sciences, Mashhad, Iran

**Keywords:** child, hydronephrosis, pyeloplasty, ultrasonography, ureteral obstruction

## Abstract

**Background/objective:**

Ureteropelvic junction obstruction (UPJO) is a common congenital anomaly in pediatric patients. Although nuclear scintigraphy is traditionally considered the standard for postoperative follow-up, the associated radiation exposure, invasiveness, and cost have prompted interest in noninvasive options. This study evaluated postoperative ultrasonographic changes as a first-line surveillance tool for children undergoing pyeloplasty.

**Methods:**

This prospective observational before-after cohort study (March 2023–March 2024) evaluated 50 children (38 girls, 12 boys) treated for primary congenital UPJO. All underwent open pyeloplasty by two experienced surgeons, with Double-J stents removed at 4 weeks. Preoperative and 3-month postoperative evaluations compared sonographic parameters [anteroposterior pelvic diameter (APD), cortical thickness, renal length, hydronephrosis severity], diethylenetriamine pentaacetic acid renal scintigraphy, and biochemical markers. Surgical success was defined as clinical improvement alongside stable or improved sonography without the need for reintervention.

**Results:**

Postoperatively, significant reductions were observed in APD, hydronephrosis grade, serum creatinine, BUN, and random urine creatinine (all *P* < 0.001). Conversely, cortical thickness and renal length significantly increased bilaterally (all *P* < 0.001). Differential renal function was effectively preserved, with no significant postoperative changes (right: *P* = 0.209; left: *P* = 0.189). Additionally, patient age demonstrated significant positive correlations with multiple postoperative sonographic and biochemical parameters.

**Conclusion:**

Ultrasonography is a safe, reliable, and practical first-line surveillance tool for post-pyeloplasty follow-up. Nuclear renography can be selectively reserved for patients showing clinical deterioration or a lack of sonographic improvement.

## Introduction

Ureteropelvic junction obstruction (UPJO) is a well-recognized clinical condition characterized by impaired urine flow from the renal pelvis into the proximal ureter. If not diagnosed and managed promptly, it can ultimately lead to irreversible renal damage and loss of function^[^1,2^]^. Most cases are congenital and are frequently detected during the second trimester of pregnancy via prenatal ultrasonography, making UPJO the leading cause of antenatal hydronephrosis, accounting for nearly 80% of cases^[^3^]^. Its prevalence is estimated at 1 in 1000 to 1 in 15 000 individuals^[^4^]^, with a higher incidence in the pediatric population. Males are twice as likely to be affected as females, and left-sided involvement is approximately twice as common as right-sided^[^3^]^. Bilateral obstruction occurs in roughly 5–10% of cases^[^5^]^. While some patients can be managed conservatively, recurrent symptoms, progressive hydronephrosis, or deterioration in renal function are the main indications for surgical intervention^[^6^]^. Pyeloplasty remains the gold-standard surgical approach, is used in a substantial proportion of cases, and is associated with an approximately 95% success rate^[^7,8^]^. The primary objective of pyeloplasty is to relieve symptoms and preserve renal function^[^9,10^]^; recurrence rates are less than 1%, and nephrectomy is rarely required^[^11^]^.

HIGHLIGHTS

- Pyeloplasty significantly reduces the anteroposterior pelvic diameter and the hydronephrosis grade in children with UPJO.
- Renal cortical thickness and kidney length increase significantly after pyeloplasty.
- Differential renal function remains stable on follow-up after surgical intervention.
- Ultrasonography is a practical first-line, noninvasive follow-up tool; renography can be reserved for clinically or sonographically concerning cases.

Diagnosis typically involves clinical assessment, ultrasonography, computed tomography, and nuclear renal scintigraphy^[^12^]^. Postoperatively, functional assessment is traditionally performed 3–6 months after surgery, with continued follow-up suggested for up to 2 years^[^13^]^. Although nuclear scans are highly effective in evaluating drainage and differential renal function (DRF), they are costly, invasive, and expose pediatric patients to ionizing radiation^[^14^]^. Consequently, many clinicians are increasingly relying on ultrasonography for postoperative monitoring, as it is safe, accessible, and highly reliable in detecting structural changes^[^15^]^. Furthermore, emerging evidence suggests that in asymptomatic patients with favorable sonographic evolution, routine postoperative diuretic renography may not always be necessary^[^16–18^]^.

A comprehensive understanding of UPJO etiology and pathophysiology is essential for effective clinical management. Differentiating congenital etiologies – such as ureteral hypoplasia, high ureteral insertion, and crossing accessory renal vessels, which account for approximately 50% of surgical cases in children – from acquired causes like retroperitoneal fibrosis or calculous disease is critical for determining the appropriate therapeutic strategy^[^3,4,19^]^. Early diagnosis of this pathology is readily achieved through prenatal ultrasonography utilizing the Society for Fetal Urology (SFU) grading system. Diuretic renography with 99mTc-DTPA (as used in the present study) remains an established reference standard for assessing DRF and the severity of obstruction^[^20–23^]^. In the broader literature, 99mTc-MAG3 is also widely used for diuretic renography, particularly for drainage assessment^[^20–23^]^. This comprehensive evaluation allows urologists to intervene promptly with pyeloplasty when renal function deteriorates to <40% or recurrent infections occur, thereby averting severe complications. Fortunately, outcomes in infants are generally favorable, and low-grade hydronephrosis (e.g., SFU grades I and II) frequently resolves spontaneously^[^24^]^.

Recent literature indicates that specific ultrasonographic parameters could serve as useful adjuncts during the post-pyeloplasty period, offering a practical and cost-effective alternative for longitudinal structural assessment. Nozarnezhad *et al* demonstrated that a 26% improvement index at 6 months post-surgery predicts surgical success with high sensitivity and specificity^[^25^]^. Similarly, Mohajerzadeh *et al* emphasized that a >26% reduction in the anteroposterior renal pelvic diameter (APD) is a strong prognostic indicator that can help reduce unnecessary diuretic renography^[^26^]^. Hamedanchi and Sedokani validated the efficacy of color Doppler ultrasonography as a noninvasive modality for confirming the resolution of obstruction^[^27^]^, while Fernández-Ibieta *et al* suggested that a >15% improvement at 3 months may be sufficient for ultrasound-based follow-up in selected patients^[^28^]^. These findings imply that establishing precise ultrasonographic thresholds may optimize postoperative monitoring while minimizing unnecessary exposure to ionizing radiation.

In this context, the present study aims to evaluate postoperative ultrasonographic changes in children with UPJO undergoing pyeloplasty at a tertiary pediatric referral center and to determine whether dynamic sonographic parameters – including APD reduction, cortical thickness, and kidney length – correlate with clinical and functional outcomes. Rather than replacing renal scintigraphy in all patients, this study seeks to clarify whether ultrasonography can identify favorable postoperative courses and help to reserve renography for selected cases of clinical concern or insufficient sonographic improvement. The study was reported in accordance with the STROCSS *2025 guidelines*^[^29^]^.

## Study design

This prospective observational before-and-after cohort study was conducted at a single tertiary pediatric referral center between March 2023 and March 2024. The study population consisted of children diagnosed with congenital UPJO who underwent pyeloplasty during the study period. A total of 50 children were included. Inclusion required complete preoperative and postoperative ultrasonographic and diethylenetriamine pentaacetic acid (DTPA) scintigraphy data. Patients were excluded if parental informed consent was not obtained; medical records were unavailable or incomplete; family follow-up could not be completed; bilateral UPJO was present; or preoperative or postoperative imaging data were missing.

## Procedures and data collection

The primary objective of this study was to evaluate postoperative ultrasonographic changes after pyeloplasty and to assess whether sonographic parameters could serve as a practical tool for follow-up in comparison with standard DTPA scintigraphy. For each patient, a comprehensive set of parameters was recorded before surgery and 3 months after surgery. Ultrasonographic measurements included renal length, APD, cortical thickness, and hydronephrosis severity. DRF was obtained from DTPA scintigraphy. Additionally, blood urea nitrogen (BUN), serum creatinine, random urine protein, and random urine creatinine were recorded.

All patients underwent open pyeloplasty performed by two experienced pediatric surgeons using a uniform operative approach. Double-J ureteral stents (3 × 12 cm or 4 × 16 cm, according to patient size and anatomy) were placed in all patients and were removed 4 weeks after surgery.

Postoperatively, patients were stratified according to sonographic response into two cohorts: those with stable or improved hydronephrosis, and those with worsened hydronephrosis. This stratification was used to describe postoperative response and to explore whether unfavorable sonographic evolution was associated with poorer clinical outcomes. For the purposes of this study, surgical success was defined as clinical improvement together with stable or improved postoperative sonographic findings and no need for reintervention, whereas surgical failure was defined as worsening hydronephrosis, persistent or recurrent obstructive symptoms, or the need for additional intervention. A clinically significant change in DRF was prospectively defined as an alteration of more than 5%.

## Statistical analysis

All statistical analyses were performed using IBM SPSS Statistics version 26.0 (IBM Corp., Armonk, NY, USA). Continuous variables are expressed as the mean ± standard deviation (SD), whereas categorical variables are reported as frequencies and percentages. Preoperative and postoperative measurements were compared using the paired-samples *t*-test. The Pearson correlation coefficient® was used to evaluate associations between continuous variables. Statistical significance was set at *P* < 0.05.

## Results

During the study period, 50 children met the inclusion criteria and were analyzed. Of these, 38 (76.0%) were female, and 12 (24.0%) were male.

As shown in Table 1, postoperative urinary protein status improved significantly. The proportion of patients with negative urinary protein increased from 20.0% preoperatively to 56.0% postoperatively, whereas the proportions of patients with trace and positive urinary protein decreased postoperatively (trace: 46.0% vs. 38.0%; 1+: 26.0% vs. 6.0%; 2+: 8.0% vs. 0%).

**Table 1:** Comparison of urine protein levels before and after surgery.

Variable	Before surgery	After surgery
Negative	10(20.0%)	28(56.0%)
Trace	23(46.0%)	19(38.0%)
+	13(26.0%)	3(6.0%)
++	4(8.0%)	0(0.0%)

As shown in Table 2, APD and hydronephrosis grade were significantly reduced in both kidneys after pyeloplasty (all *P* < 0.001). In contrast, cortical thickness and renal length increased significantly on both sides following surgery (all *P* < 0.001). DRF did not change significantly after surgery in either kidney (right: *P* = 0.209; left: *P* = 0.189).

**Table 2:** Comparison of key ultrasound indices and differential renal function before and 3 months after pyeloplasty (*N* = 50).

Variable	Before surgery (Mean ± SD)	After surgery (mean ± SD)	Pvalue
Right anteroposterior diameter (mm)	14.18 ± 6.24	8.76 ± 2.73	<0.001
Left anteroposterior diameter (mm)	14.32± 8.96	9.09 ± 4.63	<0.001
Right cortical thickness (mm)	5.70 ± 1.66	6.84 ± 1.26	<0.001
Left cortical thickness (mm)	6.05± 1.46	6.98 ± 1.32	<0.001
Right hydronephrosis grade	1.54 ± 0.95	0.76 ± 1.33	<0.001
Left hydronephrosis grade	1.20 ± 1.06	0.46± 0.73	<0.001
Right kidney length (mm)	70.08 ± 16.16	72.60 ± 15.39	<0.001
Left kidney length (mm)	72.42 ± 16.08	74.82 ± 15.78	<0.001
Right differential function (%)	53.71 ± 10.68	55.28 ± 11.69	0.209
Left differential function (%)	46.28± 10.68	44.64 ± 11.62	0.189

Data are presented as mean ± standard deviation (SD) for the entire cohort (*N* = 50). *P*-values represent paired comparisons between preoperative and 3-month postoperative measurements and were calculated using the paired-samples *t*-test. Anteroposterior pelvic diameter (APD), differential renal function (DRF), and kidney length are reported as shown in the table. DRF values were derived from DTPA renal scintigraphy. Statistical significance was defined as *P* < 0.05.

Laboratory findings also showed significant postoperative improvement. BUN, serum creatinine, and random urinary creatinine decreased significantly after surgery (all *P* < 0.001; Table 3).

**Table 3:** Comparison of biochemical and clinical parameters before and after surgery.

Variable	Before surgery (mean ± SD)	After surgery (mean ± SD)	P value*
Serum creatinine	0.55± 0.13	0.48± 0.10	< 0.001
Serum BUN	14.50± 2.68	11.92± 2.42	< 0.001
Random urine creatinine	0.36± 0.09	0.23± 0.09	< 0.001

^*^ Statistical significance defined as *P* < 0.05.

Correlation analysis between patient age and clinical variables demonstrated several significant associations (Table 4). Age showed a significant positive correlation with left APD, both preoperatively (*P* = 0.038) and postoperatively (*P* = 0.017). Age was also positively correlated with bilateral renal lengths before and after surgery (all *P* < 0.001). In addition, age was significantly and inversely correlated with postoperative random urine creatinine (*P* = 0.018).

**Table 4:** Correlation analysis between patient age and preoperative and postoperative clinical/ultrasonographic variables (*N* = 50).

Variables	Period	ρ value	Correlation coefficient®
Right anteroposterior diameter	Before surgery	0.814	0.034
	After surgery	0.243	0.168
Left anteroposterior diameter	Before surgery	0.038	0.295
	After surgery	0.017	0.336
Left cortical thickness	Before surgery	0.826	0.032
	After surgery	0.181	0.192
Right cortical thickness	Before surgery	0.606	0.075
	After surgery	0.425	0.115
Left unilateral hydronephrosis grade	Before surgery	0.147	0.208
	After surgery	0.481	0.102
Right unilateral hydronephrosis grade	Before surgery	0.268	0.160
	After surgery	0.334	0.139
Right kidney length	Before surgery	<0.001	0.829
	After surgery	<0.001	0.827
Left kidney length	Before surgery	<0.001	0.804
	After surgery	<0.001	0.804
Right kidney split function	Before surgery	0.217	0.224
	After surgery	0.588	−0.159
Left kidney split function	Before surgery	0.210	−0.228
	After surgery	0.637	0.139
Serum creatinine level	Before surgery	0.121	0.222
	After surgery	0.064	0.264
Serum BUN level	Before surgery	0.661	−0.064
	After surgery	0.661	−0.064
Random urine creatinine	Before surgery	0.204	−0.185
	After surgery	0.018	−0.336

APD, anteroposterior pelvic diameter; DRF, differential renal function. Pearson’s correlation coefficient (*r*) was used to assess the associations between patient age and the listed preoperative and postoperative clinical and ultrasonographic variables. *P*-values indicate statistical significance, defined as *P* < 0.05. “Before surgery” denotes the correlation between age at the time of pyeloplasty and the corresponding preoperative measurement; “After surgery” denotes the correlation between age at the 3-month follow-up and the corresponding postoperative measurement.

## Discussion

In this prospective observational before-and-after cohort study, postoperative evaluation demonstrated a statistically significant reduction in APD, hydronephrosis grade, serum creatinine, BUN, and random urine creatinine. In contrast, renal length and cortical thickness increased significantly after pyeloplasty. DRF remained stable, with no statistically significant change before and after surgery, suggesting preservation of baseline renal function. These findings indicate that ultrasonographic parameters reflect the structural response to pyeloplasty and may help identify patients with favorable postoperative courses.

A central issue was the role of postoperative ultrasound in relation to nuclear imaging. The present data do not justify replacing renography in all patients, nor do they provide diagnostic-performance metrics for detecting recurrent obstruction. However, the results support a more cautious and clinically realistic interpretation: in children with improving symptoms and favorable sonographic evolution, ultrasound may reduce the need for routine repeat diuretic renography, while nuclear imaging remains appropriate for patients with persistent hydronephrosis, worsening sonographic findings, or clinical concern for failure.

Several previous studies have similarly suggested that ultrasonography is a dependable and practical tool for follow-up after pyeloplasty for UPJO. Hamedanchi and Sedokani found that Doppler ultrasound effectively tracked the resolution of obstruction^[^27^]^. Similarly, Almodhen *et al* observed that none of the 101 children in their cohort with postoperative hydronephrosis required repeat surgery, highlighting the value of ultrasound in postoperative surveillance^[^30^]^. Cost *et al* emphasized its reliability, noting that only 28% of patients with recurrent hydronephrosis required reintervention within 3 months^[^17^]^. Nozarnezhad *et al* further supported this approach, suggesting that ultrasound alone may be sufficient for early follow-up, with renography reserved for cases lacking sonographic improvement^[^25^]^. In resource-limited settings, this approach is particularly relevant because ultrasonography is noninvasive, widely available, and less costly than nuclear imaging.

Among the ultrasonographic variables, APD was particularly informative. In the current study, APD decreased significantly bilaterally after surgery, supporting its usefulness as a marker of postoperative improvement. Similar reductions have been reported by Mohajerzadeh *et al*, who found that mean AP diameter decreased from 33.93 mm preoperatively to 18.1 mm at 3 months and 15.43 mm at 6 months postoperatively in 67 children^[^26^]^. El-Desoukey *et al* also reported consistent reductions in AP diameter at 3 and 6 months after surgery in children with unilateral UPJO, which is in line with our findings^[^31^]^. Prior studies have suggested that the magnitude of this reduction may have clinical value: Rickard *et al* reported that an AP diameter reduction of more than 40% is a useful indicator of pyeloplasty success^[^18^]^, while Romao *et al* showed that a 38% reduction, or a postoperative diameter of ≤11.5 mm, predicted success with 100% sensitivity^[^32^]^. Accordingly, favorable APD improvement on ultrasound may help identify patients in whom routine postoperative renography can be deferred, provided the clinical course is reassuring^[^33^]^.

The postoperative increase in renal cortical thickness also carries important clinical implications. This change likely reflects relief of obstruction and subsequent parenchymal recovery after pyeloplasty. In the present study, cortical thickness increased significantly after surgery. Chalieopanyarwong and Attawettayanon noted that renal parenchymal thickness improves after pyeloplasty, particularly in patients with relatively preserved baseline renal function^[^34^]^. Nozarnezhad *et al* and Mohajerzadeh *et al* also reported robust increases in cortical thickness following surgery^[^25,26^]^. Kim *et al* similarly described enhanced parenchymal size and thickness as indicators of improved renal tissue quality and functional recovery^[^35^]^.

The association between age and several sonographic and biochemical variables should also be interpreted in context. Older children showed greater renal length and higher left APD values, which may reflect longer disease duration, developmental renal size differences, or more advanced hydronephrosis at the time of surgery. The inverse association between age and postoperative random urine creatinine was less specific and may reflect differences in renal maturation, muscle mass, or urine concentration. Importantly, these correlations do not alter the main pre- to postoperative pattern; rather, they suggest that age may influence absolute sonographic and biochemical values.

This study has several limitations. First, the follow-up period was limited to 3 months, which may be insufficient to determine long-term postoperative stability or late recurrence of obstruction. Second, the study was conducted at a single center with a relatively small sample size, which limits generalizability. Third, while patients were stratified into stable or improved and worsened hydronephrosis groups, the current dataset does not support a formal predictive analysis of treatment failure; therefore, this stratification should be interpreted descriptively rather than as a validated outcome model. Future multicenter studies with longer follow-up and larger sample sizes are needed to validate sonographic thresholds and determine which children truly require postoperative renography.

In summary, postoperative ultrasonography demonstrated clear structural improvement after pyeloplasty and preserved DRF in children with UPJO. These findings support ultrasound as a practical first-line follow-up tool and suggest that routine nuclear scanning may be selectively reserved for patients with insufficient sonographic improvement or clinical concern for failure, rather than used uniformly in all cases.

## Conclusion

This prospective observational study demonstrates that pyeloplasty leads to significant improvements in key sonographic and biochemical parameters, specifically reductions in APD, hydronephrosis grade, serum creatinine, and random urine creatinine, along with a significant increase in renal cortical thickness and renal length; and while DRF remained stable, the structural improvements captured by ultrasonography reflect the successful relief of obstruction.

Although DTPA scintigraphy remains an established tool for assessing renal function and drainage, its routine use involves ionizing radiation exposure, higher costs, and invasiveness. Our findings suggest that in children who demonstrate favorable sonographic evolution and clinical improvement, ultrasonography can serve as a reliable, safe, and practical first-line surveillance tool for postoperative monitoring. This approach allows diuretic renography to be selectively reserved for cases that demonstrate insufficient sonographic improvement, worsening hydronephrosis, or clinical concern for surgical failure, thereby optimizing follow-up and reducing unnecessary radiation exposure in the pediatric population.

## Data Availability

The datasets used and/or analyzed during the current study are available from the corresponding author on reasonable request.

## Research registration unique identifying number (UIN)

Not applicable. This is a prospective observational before-and-after cohort study evaluating standard postoperative care outcomes, and does not meet the WHO definition of a clinical trial requiring prospective registration.
